# A Rat Model of Human Lipid Emulsion Digestion

**DOI:** 10.3389/fnut.2019.00170

**Published:** 2019-11-12

**Authors:** Andreas Steingoetter, Myrtha Arnold, Nathalie Scheuble, Shahana Fedele, Pascal Bertsch, Dian Liu, Helen L. Parker, Wolfgang Langhans, Peter Fischer

**Affiliations:** ^1^Division of Gastroenterology and Hepatology, University Hospital Zurich, Zurich, Switzerland; ^2^Department of Information Technology and Electrical Engineering, Institute for Biomedical Engineering, University and ETH Zurich, Zurich, Switzerland; ^3^Physiology and Behavior Laboratory, Department of Health Sciences and Technology, Institute of Food Nutrition and Health, ETH Zurich, Zurich, Switzerland; ^4^Laboratory of Food Process Engineering, Department of Health Sciences and Technology, Institute of Food Nutrition and Health, ETH Zurich, Zurich, Switzerland; ^5^School of Medicine, Pharmacy and Health, Durham University, Durham, United Kingdom; ^6^Institute of Health and Society, Newcastle University, Durham, United Kingdom

**Keywords:** lipid emulsion systems, fat digestion, animal model, gastric emptying, gastrointestinal hormones, satiation, energy intake

## Abstract

A better understanding of how dietary lipids are processed by the human body is necessary to allow for the control of satiation and energy intake by tailored lipid systems. To examine whether rats are a valid model of human dietary lipid processing and therefore useful for further mechanistic studies in this context, we tested in rats three lipid emulsions of different stability, which alter satiety responses in humans. Different sets of 15 adult male Sprague Dawley rats, equipped with gastric catheters alone or combined with hepatic portal vein (HPV) and vena cava (VC) catheters were maintained on a medium-fat diet and adapted to an 8 h deprivation/16 h feeding schedule. Experiments were performed in a randomized cross-over study design. After gastric infusion of the lipid emulsions, we assessed gastric emptying by the paracetamol absorption test and recorded in separate experiments food intake and plasma levels of gastrointestinal hormones and metabolites in the HPV. For an acid stable emulsion, slower gastric emptying and an enhanced release of satiating gastrointestinal (GI) hormones were observed and were associated with lower short-term energy intake in rats and less hunger in humans, respectively. The magnitude of hormonal responses was related to the acid stability and redispersibility of the emulsions and thus seems to depend on the availability of lipids for digestion. Plasma metabolite levels were unaffected by the emulsion induced changes in lipolysis. The results support that structured lipid systems are digested similarly in rats and humans. Thus unstable emulsions undergo the same intragastric destabilization in both species, i.e., increased droplet size and creaming. This work establishes the rat as a viable animal model for *in vivo* studies on the control of satiation and energy intake by tailored lipid systems.

## Introduction

Lipids are the most energy-dense macronutrients. Their overconsumption along with high sodium, sugar, and carbohydrate diets is associated with obesity, diabetes, and cardiovascular disease (1–3). A better understanding of how dietary lipids, also in relationship to carbohydrates and proteins, are processed in the human body may assist in the tailoring of lipid systems, which have the potential to modulate satiation and energy intake. Lipid digestion takes place in both the stomach and small intestine. It consists of the cleavage of the main lipid component, triacylglycerol (TAG) into monoglycerides and free fatty acids (FFA) (4). The presence of these lipid breakdown products in the stomach and the small intestine triggers the release of satiating hormones such as cholecystokinin (CCK), peptide YY (PYY), and glucagon-like peptide-1 (GLP-1) and the release of bile from the gall bladder (5–8). As these hormones also affect gastric emptying, a closed feedback loop for the control of fat digestion and absorption can be established. Most dietary lipids are ingested in the form of oil-in-water emulsions or so called lipid emulsions (LE), in which liquid oil or partially solid fat is dispersed in water (9). The ability to modify lipid digestive processing and satiation by the control of acid stability and redispersibility of the LEs has recently attracted attention (9–11).

Magnetic resonance imaging (MRI) studies in healthy subjects demonstrated that acid unstable LEs form oil layers in the upper stomach (12–14). This layering is associated with delayed gastric emptying of the lipid phase into the duodenum (15). Recent human studies addressed short-term effects of LEs on eating behavior, including the effect of intragastric LE administration on plasma CCK (16–19), active GLP-1 (17) and total PYY (19, 20). However, with oral ingestion of LEs mainly data for CCK are available (20–22). The lack of longer-term studies assessing both changes in eating behavior and GI hormones in humans is largely due to the large costs of such studies (23), associated practical limitations and high inter-individual variations. Therefore, a reliable animal model is required to assist in streamlining the development of targeted therapeutic LEs. In addition to cost saving this streamlined process may also reduce risk including adverse events to humans participating in oral application studies.

Beside pigs, rats are the most commonly used animal model for human fat digestion. Previous studies with rats addressed the effect of intragastric LE administration on eating behavior and plasma GLP-1 (24, 25), and PYY (24, 26–28). However, it remained unclear whether these findings can be related to humans. In addition, the absence of a gall bladder and gastric lipase may impede direct comparison of rat and human data (29).

In the present study in rats, we tested LEs that were previously shown to alter satiation-mediating responses in humans (30). We assessed the emulsions' effects on gastric emptying, short-term energy intake, and hepatic portal vein plasma levels of total PYY, active GLP-1 as well as TAG and compared them with human data. The aim was to examine whether the rat presents a viable animal model of human lipid digestion and processing.

## Materials and Methods

All procedures were approved by the local Veterinary Office (Protocol No. 233-2012).

### Lipid Emulsions and Layout of the Animal Studies

Fifteen adult male Sprague Dawley rats (age: 10–12 weeks, body weight: 400–500 g) were maintained on a medium-fat diet (30 kJ% fat) and adapted to a 16 h feeding 8 h deprivation schedule. The rats were equipped with hepatic portal vein (HPV), vena cava (VC) and gastric catheters. Surgeries were performed under Xylyzine/Ketamine anesthesia (4.5 mg Xylazine and 90 mg Ketamine intraperitoneally per kg body weight) (31, 32). The study was performed in a randomized cross-over design.

Three isocaloric (1.9 kcal/mL) LEs differing in acid stability, fat source, and redispersibility were intragastrically infused as single meals. The different properties and preparation schemes of the previously validated LEs are summarized in Table 1 and discussed in more detail in Steingoetter et al. and Golding et al. (14, 33). In brief, LE1 is a small-droplet acid stable emulsion. LE3 and LE4 are small-droplet acid unstable emulsions that differ from each other in their solid fat content and thus redispersibility of fat. After infusing the emulsions, gastric emptying was measured by the paracetamol absorption test and recorded food intake as well as postprandial profiles of metabolites and GI hormones in separate experiments (34).

**Table 1:** Composition and physical properties of the three lipid emulsions infused and consumed by rats and humans (LE1, LE3, and LE4 are lipid emulsion 1, 3, and 4, MG is monoglyceride, NaCas is sodium caseinate, ^*^30% of the fat was hydrogenated rapeseed oil consisting of 25% solid fat and 75% liquid fat).

	**LE1**	**LE3**	**LE4**
Fat content, weight percent	20	20	20
Fat consistency	Liquid	Solid + liquid^*^	Liquid
Emulsifier type	Polysorbate 80	NaCas + MG	NaCas + MG
Emulsifier amount, weight percent	0.8	1 + 0.25	1 + 0.25
Thickener type	Xanthan	None	None
Thickener amount, weight percent	0.4		
Mean particle size, D4,3 μm	0.33	0.32	0.38
Acid stabile	Yes	No	No
Redispersible	Yes	No	Yes

### Gastric Emptying and Food Intake

Gastric emptying and food intake were recorded in the 15 rats using the gastric catheters only. The rats were once food deprived for an additional 2 h at the onset of their 16 h feeding period and infused with 4 mL of the LEs labeled with 1% paracetamol (40 mg/4 mL). Each rat received two different LEs. Type and order of LE administration were randomized such that each LE combination was tested in five different animals. Paracetamol was measured in plasma from tail nick blood samples collected at baseline (0 min) and at 30, 60, 90, and 120 min after emulsion infusion. LE infusion days were separated by 2 washout days on which saline was infused.

For food intake measurements, the rats deprived of food for 8 h were infused with one of the three LEs 30 min immediately prior dark onset, when the 16 h food access period started. Cumulative food intake (±0.1 g) was recorded at 1, 2, 4, 6, and 16 h after infusion. Following a cross-over study design, all three LEs were tested in all rats at two different doses of 4 and 8 mL. In addition, each animal (except two) were assigned one repeated intervention. This way 43 animal food intakes were collected in total. LE infusion days were separated by one washout day.

### Plasma Concentrations of Metabolites and Gastrointestinal Hormones

For plasma concentration of metabolites and gastrointestinal hormone profiles the hepatic portal vein (HPV) and vena cava (VC) catheters in addition to the gastric catheter were used after emulsion infusion. Using the same protocol as for the paracetamol absorption test, the rats received the LE infusions, and HPV as well as VC blood was taken in parallel at baseline (0 min) and at 15, 30, 45, 60, and 120 min after emulsion infusion.

### Biochemical Analysis

Plasma paracetamol concentration was analyzed with the test kit from Cambridge Life Sciences Ltd, UK. Plasma concentrations of active GLP-1 and total PYY were measured by an electro-chemiluminescence assay (MESO Scale Discovery, USA). Plasma concentrations of TAG, FFA, and betahydroxybutyrate (BHB) were measured with enzymatic tests adapted for the Cobas Mira autoanalyzer (Cobas Mira, Hoffman La-Roche, Switzerland).

### Analysis of Emulsion Effects on Plasma Concentration Profiles of Paracetamol, Metabolites, and Gastrointestinal Hormones

Postprandial delta over baseline (DOB) curves were calculated from the plasma concentrations of paracetamol, each metabolite and gastrointestinal hormone. The area over baseline (AOB), the maximum positive (or negative) amplitude A_max_, and the time-to-maximum amplitude t_max_ were derived from these DOB curves by fitting the following power-exponential function to the data:

$$\begin{array}{l} C_{i}\left(t\right)=AOB_{i}\cdot k_{i}\cdot \beta_{i}\cdot {\left(1-e^{-k_{i}t}\right)}^{\beta_{i}-1}\cdot e^{-k_{i}t} \end{array}$$

with *i* = paracetamol, GLP-1, PYY, TAG, BHB, and FFA. The final parameter estimates t_max,i_ and A_max,i_ were calculated from t_max,i_ = log(β_i_)/k_i_ and A_max,i_ = C_i_(t_max,i_). Data fitting was achieved by Bayesian hierarchical modeling as previously specified (30). The effect of the LEs on the parameters of plasma profiles was quantified by the highest posterior density interval (HPD), also known as credible interval. The interval enclosing 95% of the posterior mass was selected. The effect sizes between the parameters are presented as median (95% HPD).

### Analysis of Emulsion Effects on Food Intake

A single logistic model was fitted to the food intake curves. The effect of intervention (emulsion vs. NaCl), emulsion type (LE1 vs. LE3 and LE4), dose (4 vs. 8 mL) and repeated record (record 1 and record 2) on the logistic model parameters asymptotic total energy intake EI_total_ in kcal and time-to-half total energy intake t_1/2_ in hours was analyzed by linear mixed effect models. “Rat” was set as random effect. Observations with absolute standardized residuals >0.995 quantile of the standard normal distribution were considered outliers and excluded for the analysis.

## Results

A total of four food intake curves, three for the study days (emulsion) and one for the washout days (NaCl) were excluded from analysis due to unstable balance readouts. A total of eight plasma concentration samples, three for gastrointestinal hormones and five for metabolites were not available due to missing data samples.

### Gastric Emptying

**Figure 1:**
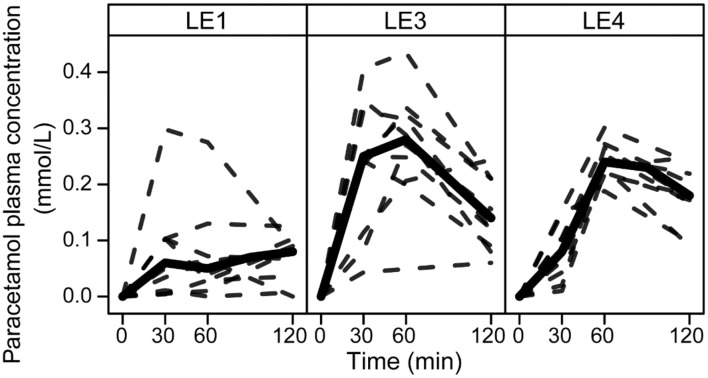
Paracetamol plasma concentration profiles in rats after gastric infusion of 4 mL of the three different LEs. Solid and dashed lines show the group median and the individual concentration curves, respectively (LE1, acid stable LE; LE3, non-redispersible acid unstable LE with solid fat; LE4, redispersible acid unstable LE).

All LEs had a distinct effect on the paracetamol plasma concentration profiles (Figure 1). A_max_ was increased for LE3 and LE4 by 0.08 mmol/L (0.05, 0.13 mmol/L) and 0.08 mmol/L (0.03, 0.1 mmol/L), respectively. Accordingly, AOB was increased for the acid unstable LE3 and LE4 compared to the acid stable LE1 by 8 mmol/L·min (3, 10 mmol/L·min) and 6 mmol/L·min (3, 8 mmol/L·min), respectively. t_max_ differed only between LE3 and LE4 with t_max_ of LE4 being later by 21 min (3, 47 min).

### Food Intake

There was an effect of intervention, emulsion type and dose on EI_total_ and t_1/2_ values. Compared to the washout days, intervention with LE1 resulted in a decrease of EI_total_ by −4.3 kcal (95% CI: −6.7, −1.8) and increase in t_1/2_ by 0.5 h (95% CI: 0.2, 0.6). Intervention with LE3 had no effect on EI_total_ [−1.4 kcal (95% CI: −3.8, 0.9)], but decreased t_1/2_ by −0.2 h (95% CI: −0.4, −0.03). Intervention with LE4 had neither an effect on EI_total_ [−1.3 kcal (95% CI: −3.4, 1.1)] nor t_1/2_ [−0.05 h (95% CI: −0.2, 0.1)]. Table 2 lists the effects of acid unstable vs. acid stable emulsions, doubling the dose and repetition of the intervention on the values for EI_total_ and t_1/2_. For LE3 and LE4 an increase of EI_total_ and decreased t_1/2_ was observed. The 8 mL dose had no effect on EI_total_, but decreased t_1/2_. The second records showed an increase in the EI_total_ and accordingly a decrease in the t_1/2_. Neither emulsion type nor dose nor record modulated the EI_total_ or t_1/2_ values of the subsequent washout days.

**Table 2:** Effect sizes for the emulsion dependent energy intake in rats after gastric infusion of the three different LEs (Effect sizes with the 95% CI interval not including zero are labeled with^*^).

		**Effect sizes, mean (95% CI)**				
	**Parameter**	**LE1 (reference)**	**LE3**	**LE4**	**8 mL dose**	**2nd record**
Emulsion intervention	EI__total_, kcal	99 (95, 103)	3.4 (1.5, 5.2)^*^	3.2 (1.1, 4.9)^*^	0.7 (−0.8, 2.3)	2.0 (0.3, 3.7)^*^
	t_1/2_, h	4.6 (4.3, 4.9)	−0.6 (−0.8, −0.5)^*^	−0.4 (−0.6, −0.2)^*^	−0.3 (−0.4, −0.1)^*^	−0.2 (−0.3, −0.05)^*^
Subsequent washout days	EI__total_, kcal	103 (101, 106)	−0.7 (−2.2, 0.5)	−1.1 (−2.5, 0.3)	−0.9 (−1.9, 0.3)	−0.3 (−1.5, 0.9)
	t_1/2_, h	4.2 (4.0, 4.3)	0.05 (−0.02, 0.1)	0.06 (−0.01, 0.1)	0.04 (−0.03, 0.1)	0.02 (−0.04, 0.09)

### Plasma Concentrations of Gastrointestinal Hormones and Metabolites

**Figure 2:**
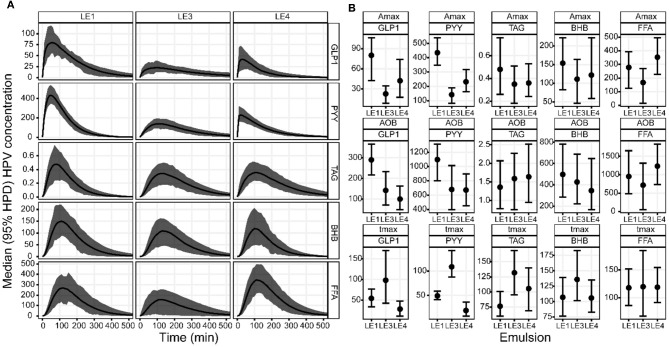
**(A)** Group median DOB curves and **(B)** parameter estimates with 95% HPD of the GLP-1, PYY, TAG, BHB, and FFA plasma concentration profiles in the hepatic portal vein (HPV) in rats after gastric infusion of 4 mL of the three different LEs. The DOB curves are grouped by LE (columns) and blood measure (rows). The concentration profiles have units ρg/mL for GLP-1 and PYY, mmol/L for TAG, μmol/L for BHB and FFA. The boxplots are grouped by parameter (rows) and blood measure (columns). The values for Amax are equal to the respective concentration profiles. The values for AOB are ρg/mL·h for GLP-1 and PYY, mmol/L·min for TAG, μmol/L·min for BHB and FFA. The value of t_max_ is given in min.

**Figure 3:**
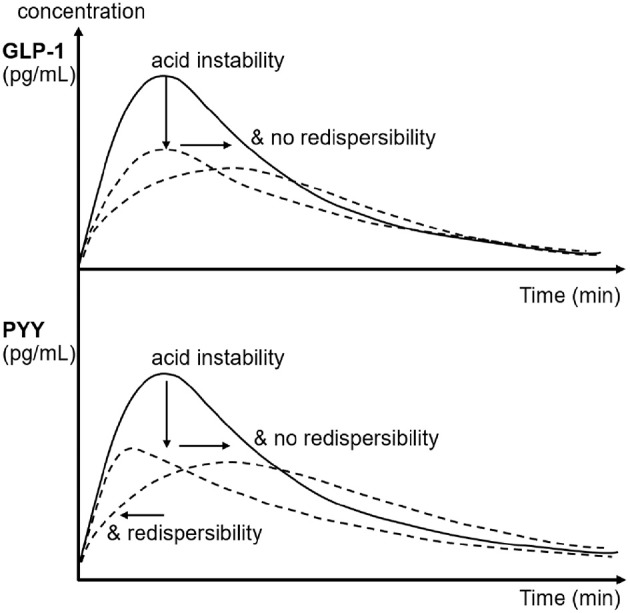
Schematic of the effects of emulsion stability and redispersibility on plasma concentration profiles of GLP-1 and PYY in rats after gastric infusion of 4 mL of the three different LEs. The arrows indicate the decrease and shift in peak concentration due to the changes in emulsion stability and redispersibility.

Visual inspection of the DOB curves and boxplots in Figures 2A,B indicate that the hormone and metabolite profiles have a similar response to the individual LEs, especially regarding peak height and relative timing. This LE dependency was clearly compromised by the large inter-individual variation observed for the metabolite profiles. LEs had the most distinct effect on the profiles of PYY (Figure 3). This was equally well-detectable in the blood of HPV and VC (Table 1). A_max_ and t_max_ of PYY were different among all three LEs. A_max_ was highest for LE1 and lowest for LE3. t_max_ was shortest for LE4 and longest for LE3. AOB of PYY was lower for LE3 and LE4 compared to LE1. The LEs also had differential effects on GLP-1 profiles (Figure 3). This was better reflected in HPV than in VC blood plasma (Table 3). A_max_ and AOB of GLP-1 were lower for LE3 and LE4 compared to LE1. t_max_ was shorter for LE4 than LE3. Postprandial TAG and BHB plasma concentration showed similar patterns for all LEs and differed only in a longer t_max_ for LE3 than LE1. Postprandial FFA plasma concentrations did not exhibit any systematic changes, and no effect of LEs neither for HPV nor VC blood were detected.

**Table 3:** Effect of acid stability and redispersibility on gastrointestinal hormone profiles in rats after gastric infusion of 4 mL of the three different LEs (only parameters exhibiting effect sizes with 95% HPDs not including zero are listed in this table.

**Hormone**	**Blood**	**Parameter**	**Median (95% HPD)**	**Emulsion effect**
GLP-1	HPV	ΔA_max_31, *pg/mL*	−57 (−87 to −28)	Acid instability (LE3, LE4)
		ΔA_max_41, *pg/mL*	−37 (−72 to −2)	→ lower GLP-1 peak
		Δt_max_43, *min*	−68 (−142 to −21)	Acid instability and no redispersibility (LE3)
				→ delayed GLP-1 peak
		ΔAOB31, *pg/mL·h*	−155 (−232 to −25)	Acid instability (LE3, LE4)
		ΔAOB41, *pg/mL·h*	−194 (−256 to −114)	→ less GLP-1 release
	VC	ΔA_max_31, *pg/mL*	−19 (−28 to −11)	see HPV
		ΔA_max_41, *pg/mL*	−17 (−26 to −10)	see HPV
		ΔAOB41, *pg/mL·h*	−39 (−59 to −13)	Acid instability and redispersibilty (LE4)
				→ less GLP-1 release
PYY	HPV	ΔA_max_31, *pg/mL*	−303 (−374 to −222)	Acid instability (LE3, LE4)
		ΔA_max_41, *pg/mL*	−202 (−283 to −131)	→ lower PYY peak.
		ΔA_max_43, *pg/mL*	91 (30 to 162)	No redispersibility (LE3) → further decrease in PYY peak
		Δt_max_31, *min*	60 (34 to 87)	Acid instability and no redispersibility (LE3)
				→ delayed PYY peak
		Δt_max_41, *min*	−30 (−44 to −11)	Acid instability and redispersibility (LE4)
		Δt_max_43, *min*	−90 (−123 to −65)	→ earlier PYY peak
		ΔAOB31, *pg/mL·h*	−418 (−703 to −94)	Acid instability (LE3, LE4)
		ΔAOB41, *pg/mL·h*	−437 (−737 to −228)	→ less PYY release
	VC	ΔA_max_31, *pg/mL*	−240 (−332 to −166)	see HPV
		ΔA_max_41, *pg/mL*	−166 (−266 to −108)	see HPV
		ΔA_max_43, *pg/mL*	66 (8 to 116)	see HPV
		Δt_max_31, *min*	65 (35 to 123)	see HPV
		Δt_max_41, *min*	−26 (−47 to −4)	see HPV
		Δt_max_43, *min*	−94 (−142 to −50)	see HPV
		ΔAOB31, *pg/mL·h*	−354 (−579 to −104)	see HPV
		ΔAOB41, *pg/mL·h*	−331 (−544 to −163)	see HPV
**TAG**	HPV	Δt_max_31, *min*	53 (12 to 99)	Acid instability and no redispersibility (LE3)
**BHB**	VC	Δt_max_31, *min*	35 (0.5 to 76)	→ delayed TAG and BHB peak

*ΔAmax41, Δtmax41, ΔAOB41 are the effect sizes of the maximum amplitude (A_max_), the time-to-maximum amplitude (t_max_) and area over baseline (AOB) between the acid unstable LE4 and the acid stable LE1. All other effect sizes are encoded accordingly. 95% HPD: 95% highest posterior density interval. LE1: acid stable LE; LE3: non-redispersible acid unstable LE with solid fat, LE4: redispersible acid unstable LE, CCK: cholecystokinin, PYY: peptide YY, GLP-1: glucagon-like peptide 1, BHB: betahydroxybutyrate, HPV: hepatic portal vein, VC: vena cava.)*.

### Comparison of Rat and Human Data

**Figure 4:**
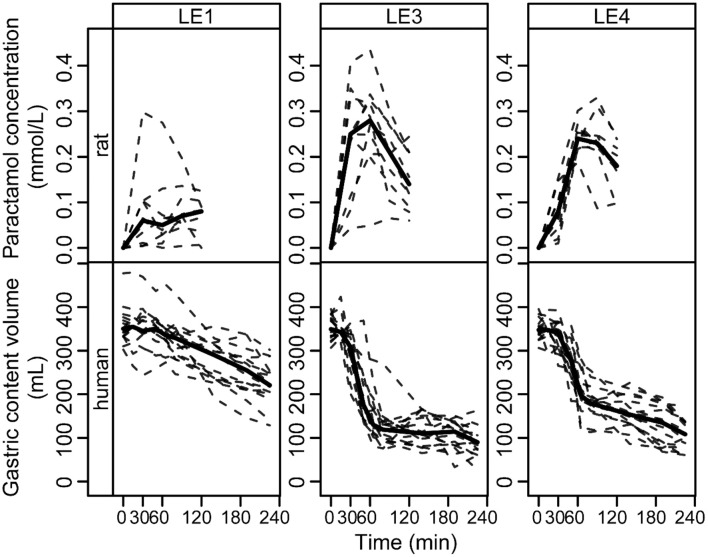
Paracetamol plasma concentration profiles in rats and gastric content emptying curves in humans after intake of the three different LEs. The data is grouped by LE (columns) and species (rows). Solid and dashed lines show the group median and individual curves, respectively.

**Figure 5:**
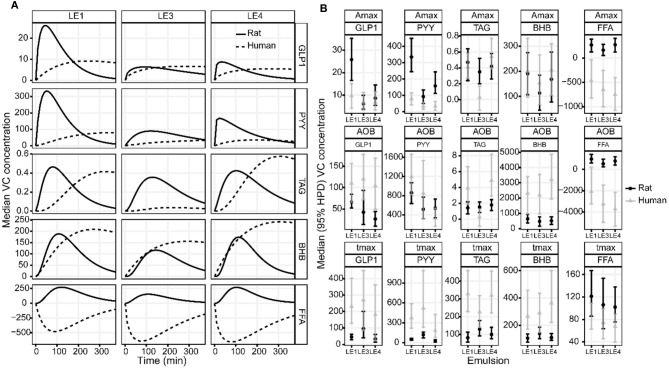
**(A)** Estimated group average DOB curves and **(B)** parameter estimates of the GLP-1, PYY, TAG, BHB, and FFA plasma concentration profiles in rats and humans after intake of the three different LEs. **(A)** The DOB curves are grouped by LE (columns) and blood parameter (rows). Solid and dashed black lines indicate group average rat and human DOB curves, respectively. The concentration profiles have units ρg/mL for GLP-1 and PYY, mmol/L for TAG, μmol/L for BHB and FFA. **(B)** The boxplots are grouped by parameter (rows) and blood measure (columns). The values for Amax are equal to the respective concentration profiles. The values for AOB are ρg/mL·h for GLP-1 and PYY, mmol/L·min for TAG, μmol/L·min for BHB and FFA. The value of t_max_ is given in min.

The LE dependent gastric emptying dynamic in rats (Figures 1–3) is clearly recognizable in human data despite the very different measurement approaches, i.e., indirect marker technique by paracetamol (rats) vs. direct volume assessment by MRI in humans (Figure 4): The acid stable LE1 showed slow and steady emptying. In contrast, the acid-unstable LE3 and LE4 showed immediate emptying. The maximum hormonal response was reached earlier in rats than in humans (Figure 5A). Nevertheless, the LE dependency of plasma hormone levels was in good agreement between rats and humans. PYY and GLP-1 responses were greatest for LE1 and lowest and most delayed for LE3 (Figure 5B and Table 4). Further, the effect on food intake in rats are consistent with patterns in the emulsion-dependent postprandial hunger and fullness scores of the human subjects (30). Subjects reported lowest hunger and highest fullness scores for LE1, which agrees with the lowest EI_total_ and longest t_1/2_ in rats for this emulsion.

**Table 4:** Comparing the effects of emulsions stability and redispersibility on gastrointestinal hormone profiles between rats and humans after intake of the three different LEs (LE1, acid stable LE; LE3, non-redispersible acid unstable LE with solid fat; LE4, redispersible acid unstable LE; CCK, cholecystokinin; PYY, peptide YY; GLP-1, glucagon-like peptide 1; BHB, betahydroxybutyrate; FFA, free fatty acids).

**Measure**	**Effect**	**Rat**	**Human**	
GLP-1	ΔA_max_31, pg/mL	−19 (−28 to −11)	−3.0 (−6.4, 0.2)	Acid instability (LE3, LE4)
	ΔA_max_41, pg/mL	−17 (−26 to −10)	−2.6 (−5.4, 0.5)	→ lower GLP-1 peak in rats and in humans
	ΔAOB41, pg/mL·h	−39 (−59 to −13)	−3 (−57, 75)	Acid instability and redispersibility (LE4)
				→ less GLP-1 release only in rats
PYY	ΔA_max_31, pg/mL	−240 (−332 to −166)	−47 (−79, −19)	Acid instability (LE3, LE4)
	ΔA_max_41, pg/mL	−166 (−266 to −108)	−44 (−79, −15)	→ lower PYY peak in rats and humans
	Δt_max_41, min	−26 (−47 to −4)	−178 (−369, −39)	Acid instability and redispersibility (LE4)
	Δt_max_43, min	−94 (−142 to −50)	−336 (−802, −31)	→ earlier PYY peak in rats and humans
	ΔAOB31, pg/mL·h	−354 (−579 to −104)	−343 (−912, 522)	Acid instability and no redispersibility (LE3)
				→ less PYY release only in rats
	Δt_max_31, min	65 (35 to 123)	151 (−132, 599)	Acid instability and no redispersibility (LE3)
				→ delayed PYY peak only in rats
TAG	Δt_max_31, min	53 (12 to 99)	−146 (−352, 11)	Acid instability and no redispersibility (LE3)
				→ earlier TAG peak only in rats
	dA_max_31	−0.1 (−0.2, 0.1)	−0.4 (−0.6, −0.2)	Acid instability and no redispersibility (LE3)
	dAOB31	4 (−35, 47)	−236 (−439, −105)	→ lower TAG peak and release only in humans
BHB	Δt_max_31, min	35 (0.5 to 76)	42 (−169, 216)	Acid instability and no redispersibility (LE3)
				→ delayed BHB peak only in rats
FFA				No emulsion effect neither in rats nor in humans

## Discussion

Three LEs with different gastric stability were tested in randomized cross-over studies to establish a human-rat model for dietary lipid digestion. In both species food intake and release of satiating GI hormones was correlated to the design of the LEs, thus providing a link between food structure and fat digestion. This animal study demonstrates that an acid stable lipid emulsion (LE1) increased satiating responses including plasma GLP1 and PYY release. It also reduced gastric emptying rates compared with acid unstable emulsions (LE3, LE4). These physiological findings are consistent with previously published (30) human data. Thus, even though the same amount of fat was administered with each single emulsion, the physiological responses in rats and humans differed in similar fashion. The results in both rats and humans can be attributed to differences in emulsion structure formed during digestion as previously observed *in vitro* (33, 35) and *in vivo* (14, 15, 36). The magnitude of the hormonal responses was associated to the acid stability and redispersibility of the emulsions and thus likely to be dependent on the availability of lipids for digestion (LE1 > LE4 > LE3 in decreasing order). Rats may therefore be a viable model for gastrointestinal human fat processing.

We assessed gastric emptying by measuring the paracetamol plasma concentration after gastric infusion of the LEs. Paracetamol is a hydrophilic compound known for its fast and complete absorption. Its plasma level therefore represents the emptying rate of the aqueous phase of LEs (37). LE1 exhibited the slowest increase in paracetamol concentration indicating simultaneous emptying of the aqueous and lipid phase as can be expected for an acid stable emulsion. The steady release of small fat droplets into the small intestine is known to slow gastric emptying (13, 38). The same emptying characteristics of LE1 were previously identified in humans (14, 15).

The acid-unstable emulsions LE3 and LE4 exhibited an instantaneous and rapid increase in paracetamol plasma concentration with peak concentrations at 30 and 60 min, respectively. Both LEs destabilize in the gastric environment resulting in an increased droplet size and hence a decreased specific surface area resulting in a reduced rate of lipolysis (39, 40). The previous human MRI study confirmed that LE3 forms indispersible semi-solid fat particles whilst LE4 forms a creamed redispersible lipid layer inside the stomach (14). These processes lead to a rapid emptying of the total gastric content which includes both the faster emptying of the aqueous phase and fat phase (15). The semi-solid state of LE3 is most likely an additional factor that impaired lipolysis kinetics (41). Kalogeris et al. observed that gastric emptying rate is inversely correlated with meal nutrient density in rats (42) and results from this study indicate that a similar effect can be achieved by different food structuring. Based on our lipid emulsion systems we showed that in agreement with human studies LE structuring alters gastric emptying rates in rats.

The effect of LE stability on the satiating hormones GLP-1 and PYY was analyzed by sampling blood from both the HPV and VC. This challenging procedure was motivated by the fact that intact GLP-1 is rapidly degraded during passage across the hepatic bed by DPP IV associated with hepatocytes (43). A previous study in rats showed that the GLP-1 concentration in the HPV was always higher than in the VC (44). Nevertheless, the effect of LE stability on GLP-1 concentration profiles was detectable in plasma from both blood vessels. Hence hepatic portal vein catheterization is not strictly required for the detection of LE-related effects on GLP-1 concentrations. Moreover, the good detectability of LE effects even at lower concentrations may be attributed to the numerically robust Bayesian hierarchical fit procedure. This allowed for the extraction of dynamic features of plasma profiles, which are otherwise impossible to detect using the widely applied area under the curve (AUC) measure.

The hormonal plasma concentration profiles revealed that the acid stable emulsion LE1 stimulated the greatest release of GLP-1 and PYY. This is likely due to the constant emptying of the small fat droplets into the duodenum resulting in the highest rate of lipolysis and subsequent fatty acid sensing. LE3 caused delayed GLP-1 and PYY concentration peaks and a smaller overall hormone release compared to LE4 and LE1. This may be attributed to its semi-solid state impairing lipolysis and thus fatty acid sensing. There are no previous studies that analyzed the effects of emulsion stability and emptying on satiating hormones in rats. Lipolysis kinetics were, however, described to depend on lipid droplet size also in rats (45). Furthermore, lipid induced gastrointestinal hormone release has been previously investigated (26, 46). An increase in plasma PYY after intraduodenal and intraileal oleic acid administration to rats was reported. These findings using oleic acid administration, however, contrast results from experiments using the vascularly perfused rat ileum or colon (24, 25, 27). These indicated that there was no hormone response upon perfusion with oleic acid, but a response was noted with the bile salt taurocholate. The authors postulated that an indirect GLP-1 and PYY release mechanism involving bile salts could provide one possible explanation. Alternatively, it may be relevant whether the enteroendocrine cells are exposed to oleic acid from the intestinal lumen or from the blood.

The observed LE-dependent satiating hormone responses and gastric emptying patterns fit the lipid emulsion effects on short-term food intake. Rats consumed less food after infusion of LE1 compared to LE3 and LE4. Interestingly, however, circulating fat metabolites were only marginally influenced by emulsion stability. TAG and BHB plasma peak concentrations were slightly delayed for LE3, but no emulsion effect was found for FFA plasma concentrations. Both findings are in agreement with previous observations in humans (47). This could indicate that fat metabolism in relation to rapeseed oil is largely uncoupled from lipolysis kinetics, which in turn suggests that mainly gastric emulsion stability modulates gastric emptying rate and lipolysis kinetics in rats. These modulations may influence hormonal responses directly by fatty acid sensing or also indirectly by the release of other substances such as bile salts.

The observed effects of emulsion stability on rat gastrointestinal physiology were largely in agreement with the effects previously observed for humans. To allow for a more detailed visual comparison, rat and human plasma concentration profiles and gastric emptying curves were overlaid or displayed side-by-side (Figures 4, 5). Only VC rat blood measures are shown for better comparison with humans. Gastric emptying in humans and rats followed similar patterns, even though in rats it was measured indirectly via the paracetamol plasma concentrations. The acid stable LE1 showed slow and steady emptying, while the acid unstable LE3 and LE4 showed immediate emptying. Thus, acid unstable emulsions undergo the same destabilization in both rats and humans, i.e., increased droplet size and creaming. Also, plasma hormone levels were in good agreement between rats and humans. PYY and GLP-1 responses were largest for LE1, indicating increased lipolysis kinetics due to smaller lipid droplets. LE3 resulted in a delayed satiating response. This can probably be attributed to formation of semi-solid fat particles that are not redispersible and delay lipolysis. Although inter-emulsion responses were similar in rats and humans, corresponding plasma concentration profiles differed in dynamics. The maximum response was reached earlier in rats than in humans, which is most likely due to their overall faster metabolism. In humans, plasma TAG concentrations were significantly lower for LE3 compared to LE1 and LE4. This finding was not supported in the rat study, indicating that intraluminal lipolysis of LE3 and enterocyte reesterification of fatty acids are more efficient in rats than in humans. This may originate from a superimposed effect of an overall faster metabolism and smaller semi-solid fat particles generated in rats resulting in a higher surface to volume ratio easing luminal lipolysis. Such smaller fat particles might derive from the lower fat amount administered or higher shear forces generated in the rat stomach due to the smaller pylorus. Generally, more effects of emulsion type on hormonal responses were detected in rats. This may be explained by (i) the smaller inter-individual differences, (ii) the more central location of blood sampling, and (iii) a more robust modeling of the faster hormone profile response in rats. Interestingly, postprandial FFA increased in rats, which is presumably because some fatty acids escaped the lipoprotein lipase-mediated uptake of TAG into tissues, whereas FFA partly decreased in humans. This decrease in FFA, however, is commonly observed in humans after breakfast and presumably due to an insulin-induced inhibition of lipolysis from adipose tissue (48).

Overall the dynamics in rats and humans showed surprisingly similar characteristics for both rats and humans. Key satiating physiological signals specifically modulated by these systems show similar characteristics with differences most prominent in timing of peak amplitudes and clearance times. Given the study hypothesis and design, the underlying reasons and mechanisms for these differences cannot be investigated within this study design. However, changes in short-term energy intake in rats reflect the changes in self-reported sensations of hunger and fullness in humans. These findings therefore support the use of rat models for reliable and relevant *in vivo* studies on the control of satiation and energy intake by tailored lipid systems.

## Data Availability Statement

All datasets generated for this study are included in the article/supplementary material.

## Ethics Statement

The animal study was reviewed and approved by Kanton Zurich, Veterinary Office, Protocol No. 233-2012.

## Author Contributions

AS, MA, HP, WL, and PB designed research. MA, SF, NS, and HP conducted research. MA, AS, and DL analyzed data. AS and DL performed statistical analysis. AS, NS, PB, DL, and PF wrote paper. AS, PF, and WL had primary responsibility for final content. All authors read and approved the manuscript.

### Conflict of Interest

The authors declare that the research was conducted in the absence of any commercial or financial relationships that could be construed as a potential conflict of interest.
